# What Is the Relationship between Dopamine and Effort?

**DOI:** 10.1016/j.tins.2018.10.001

**Published:** 2019-02

**Authors:** Mark E. Walton, Sebastien Bouret

**Affiliations:** 1 Department of Experimental Psychology, University of Oxford, Tinsley Building, Mansfield Road, Oxford OX1 3SR, UK; 2 Institut du Cerveau et de la Moelle Épinière (ICM), Centre National de la Recherche Scientifique (CNRS), Hôpital Pitié Salpêtrière, 75013 Paris, France

**Keywords:** electrophysiology, voltammetry, cost–benefit decision making, motivation, midbrain, striatum

## Abstract

The trade-off between reward and effort is at the heart of most behavioral theories, from ecology to economics. Compared to reward, however, effort remains poorly understood, both at the behavioral and neurophysiological levels. This is important because unwillingness to overcome effort to gain reward is a common feature of many neuropsychiatric and neurological disorders. A recent surge in interest in the neurobiological basis of effort has led to seemingly conflicting results regarding the role of dopamine. We argue here that, upon closer examination, there is actually striking consensus across studies: dopamine primarily codes for future reward but is less sensitive to anticipated effort cost. This strong association between dopamine and the incentive effects of rewards places dopamine in a key position to promote reward-directed action.

## Highlights

Compared to reward, effort remains poorly understood, both at the behavioral and neurophysiological levels.

Dopamine has been proposed as central to effort-related decision making, but its role is not clearly defined.

In fact, the activity of midbrain dopamine neurons and mesolimbic dopamine levels are consistently modulated by anticipated future reward more strongly and consistently than effort, even when the weight of reward and effort on behavior are equated.

These signals may promote decisions to act based on the potential gain from a future reward.

## Dopamine, Benefits, and Costs

Dopamine plays a central role in reward-guided learning and motivation. In general terms, there is a broad agreement on its role in appetitive motivation, namely that dopamine mediates the positive influence of potential future rewards on behavior (action intensity, approach, learning, and decision making) 1, 2, 3, 4, 5, 6, 7, 8, 9. In particular, a recurring theme is that normal dopamine transmission is necessary to activate organisms, which in turn allows them to exert effort and gain positive outcomes [10].

However, beyond this initial consensus, there is much debate over the precise relationship between dopamine – particularly the rapid and transient (‘phasic’) changes in dopamine observed when animals are presented with or approach response options – and effortful choices. Part of the reason is that, to date, experiments that directly investigated effort-based decision making and dopamine have not always measured the separable influences of effort and reward. For instance, in many situations, effort has been studied in paradigms where the amount of force produced conjointly scaled with the amount of reward (e.g., [11]). In other experiments, subjects could earn more or better rewards by exerting more effort (e.g., 12, 13). In these experiments one can only assess the relative sensitivity to effort/reward; effort cannot be disentangled from reward because increases in difficulty can be compensated by increase in reward. Moreover, to be able to draw conclusions about the effect of different economic parameters on dopamine, it is vital that parameters are conjointly quantified and controlled 14, 15, 16, 17. This is particularly important when different studies require animals to overcome different effort demands (the varieties of effort cost are discussed in Box 1).

Box 1 **Not All Effort Is Equal**

In neuroscientific experiments investigating effort-related choice, effort is usually treated as a cost which can diminish preference for rewards or other goals that an organism may find more desirable [75]. Perhaps not surprisingly, animals have been shown to be very sensitive to energy demands and metabolic state when foraging 52, 76, 77. In rodent studies of effort-related decision making, the two most common ways of loading an effort cost have been, on a maze, to place a physical barrier between the start point of the rodent and a reward or, in an operant chamber, to impose a lever-press requirement to obtain the reward 78, 79. In monkeys and humans, studies have tended to use either repeated actions, as in some rodent studies, or a hand-held grip that requires a particular force to be produced 11, 18, 30. More recently, there have also been attempts to examine other forms of effort that are independent of changes in reward rate, and which tend to fall under the general rubric of ‘cognitive’ effort 80, 81, 82. All these types of manipulation can systematically alter the choices of the animals, with higher costs resulting in lower preference for the respective option over a lower-effort alternative.

However, the precise nature of these effort costs is not straightforward to isolate, quantify, and compare across studies. For instance, preferences for scaling a physical barrier can be temporarily reduced by inducing physical fatigue [83], suggesting that this cost may be related to exertion. Perhaps mitigating this caveat, however, is the fact that the energy requirements of climbing and jumping in small animals such as rodents are likely negligible compared to other homeostatic needs ([84] and A. Kacelnik, personal communication), suggesting that a barrier imposes more than a simple physiological cost. The effort involved in repeated responding is even less clear, particularly because this usually involves responding over longer periods of time (note, however, that several studies have demonstrated that changes in effort-related choice cannot be simply explained by an increased delay to reward or change in reward rate 85, 86). Moreover, effort costs may differentially influence distinct elements of motivation. For instance, a high response schedule can change the likelihood of initiating an action but not influence simple Pavlovian responses such as appetitive lipping by a monkey in anticipation of the upcoming delivery of water 36, 87. Equally, pharmacological manipulations can leave patterns of schedule length-related choice relatively unaltered while modulating the amount of force that is produced [88].

These distinctions are important not only when considering the normative decision-making strategy in different model organisms but also because there is increasing evidence that the neural circuits necessary to assess and overcome different types of effort cost are partially distinct. For instance, whereas depletion of nucleus accumbens dopamine can cause animals to be effort-averse when choosing whether or not to scale a barrier or when needing to make multiple lever presses, they have little effect on choices between options with different force requirements [89].

Alt-text: Box 1

Another reason for the conflicting interpretations about the relationship between dopamine and effort is that it has not always been clearly specified at which points during an effort-based decision dopamine might be acting. The reduction in willingness to overcome effort constraints observed following pharmacological manipulations could result from at least three factors: (i) changes in the way costs and benefits are valued or compared, (ii) the willingness to initiate a choice, and/or (i) the motivation to overcome the cost (of note, there might also be changes in how subjects learn about effort, but given that data on this issue are currently lacking, it will not be discussed further here).

To make matters even more complicated, there have been misunderstandings about how to interpret those existing data that do directly speak to the issue of the relationship between dopamine and effort. We have undertaken studies to systemically examine how both reward and effort influence dopamine activity [18] and release 19, 20. Despite using different techniques in different species at different levels of the dopamine system, we have obtained – in our opinion – strikingly convergent results. Nevertheless, we have found our data being used, respectively, to argue for effort coding and effort insensitivity of dopamine, sometimes even in the same article 21, 22.

Motivated by these discrepancies, controversies, and conflicting interpretations, we review here the evidence for a role for phasic changes in dopamine during effort-related decision making. We outline areas where there seems to be a consensus and areas where disagreements remain. Finally, we address the open questions that we see as being crucial for further progress in this area.

## The Influence of Upcoming Effort on Dopamine Neuronal Activity

There is an extensive literature, both in primates and rodents, showing that the responses of many midbrain dopamine neurons elicited by predictive cues reflect the expected value of a future reward. Such signals are consistently larger when the benefits will be greater 18, 23, 24, 25, but can also be negatively influenced by costs that directly relate to a decrease in reward availability, such as the delay in that future reward or its probability 24, 26, 27, 28, 29. This coding also incorporates individual preferences for different reward types, and can be modulated by risk inclination, fatigue, and satiety 16, 18, 30, 31. Not surprisingly, this rich set of findings has been used to support a position that dopamine activity encodes a subjective value, or utility, signal that is appropriate for driving decision making [32]. Of note, these data were obtained in well-trained animals because variability in behavior in early phases of training makes the interpretation of the relationship between brain activity and behavior more difficult. Crucially, however, the animals in these experiments showed sufficient behavioral flexibility to rule out an interpretation in terms of habit or overtraining. Instead, they mastered the task rules well enough to use information appropriately to make decisions that minimize costs and maximize benefits.

However, because we all know from daily life situations (for instance, whether or not to run to catch a train) that any decision relies not only on the anticipated value of future reward but also on predictions of the effort that must be exerted to gain this benefit (Box 2 for consideration of how to define a cost). Therefore, if dopamine is to be considered a suitable signal to drive economic choice – in other words, if dopamine represents a net utility signal – it must also be modulated by anticipated effort costs. However, to date, the evidence for this is lacking.

Box 2 **Effort: A Special Type of Cost?**

The trade-off between costs and benefits is central to many disciplines interested in behavior. In economics, decision making is captured by a comparison between the utility of potential items (goods or services) on offer ([90] for review). The utility of an item increases with the expected benefits (the size of a reward, for instance), and decreases with the expected costs. In that frame, any feature that leads to a decrease in utility can be considered as a cost: the effort required to obtain it, the delay until it is obtained, or the risk of not obtaining it (if it is chosen). Optimal decision making occurs here when the choice is based on a perfect evaluation of the (expected) utility of the items to be chosen from. In other words, what is maximized is the precision with which the function that relates the objective measures (reward size, effort, delay, risk) maps onto the subjective value of an individual, as measured using choices.

In biology, the trade-off between costs and benefits is also central, but what animals are thought to optimize in their behavior is the allocation of energy, rather than utility. This has been captured by the theory of optimal foraging, which predicts that the energy spent to obtain food should not exceed the energy provided by the food obtained 77, 91. Note that animals must also solve life-history trade-offs, for instance between reproductive costs (mating, parenting) and somatic efforts (growth, storage, maintenance) 92, 93. Over time, animals are thought to maximize the rate of energy intake and minimize their energy expenditure to maintain a positive energy balance. Crucially, this balance between energy costs and benefits could be achieved through two formally distinct (but not exclusive) strategies: (i) a selection of appropriate behaviors through evolution, which implies that such behaviors would be both relatively fixed for a given species and adaptive in their natural environment, and (ii) a cognitive evaluation of potential costs and benefits associated with a given action, which would allow finer adaptation to individual choices. In the latter case, effort costs correspond to the anticipated energy expenditure per unit time of the potential actions, allowing planning of upcoming behavior. Various species presumably display distinct combinations of these strategies, and therefore different ways to adjust their energy balance as a function of the ecological constraints in which they evolved (94, 95, 96, 97, 98, 99, 100 and Louail *et al*., unpublished).

Alt-text: Box 2

**Figure 1:**
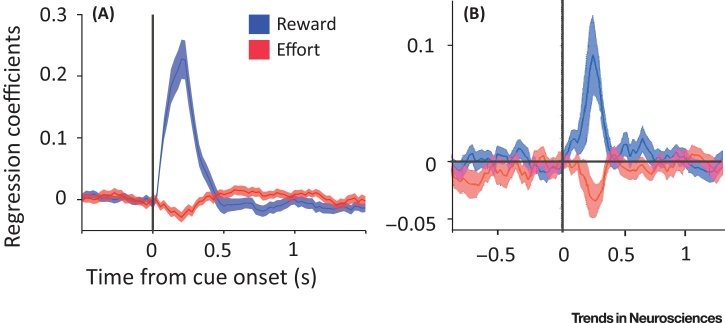
Relative Sensitivity of Dopamine (DA) Neurons to reward Benefits and Effort Costs. Sensitivity of substantia nigra pars compacta dopamine neurons in monkeys to information about upcoming reward benefits (blue) and effort costs (red) in two recent neurophysiological studies (A,B) in behaving monkeys. In both studies, monkeys were required to perform a given action to obtain a given reward. Reward sizes and physical difficulty (effort cost) were manipulated independently across trials, and each trial started with a visual cue indicating the upcoming effort and reward. Regression coefficients were calculated using a sliding-window procedure to evaluate the difference in firing across reward (blue) and effort (red conditions) at each time-point around stimulus onset. The firing of dopamine neurons shows reliable positive encoding of reward size (firing rates are greater for cues indicating large versus small rewards) within 200 ms after cue onset. At the same time, dopamine neurons also display negative modulation by effort level (firing rates are smaller for larger effort levels). Crucially, the magnitude of the reward modulation is greater than the effort modulation in both studies, even though they clearly differ in the way animals needed to cope with the expected difficulty. The difference in sensitivity cannot be simply due to a difference in subjective sensitivity to reward, as compared to effort, because these two variables had an equivalent weight on the willingness to work of the animal, at least in [18]. Panel (A) reproduced, with permission, from [30]; panel (B) adapted, with permission, from [18].

One example comes from a reward schedule task where monkeys have to complete a series of between 1 and 4 correct trials to gain reward, signaled by a predictive cue indicating the number of trials remaining in the schedule. In these experiments, the majority of dopamine neurons showed no sensitivity to cued information about schedule length, even though this information had a strong influence on the motivation of the monkey to perform the task (i.e., monkeys were much more likely to engage in the task for short compared to long schedules) [33]. Notably, the strong but equivalent levels of dopamine activity to the first cue in each sequence was mirrored by an equivalent level of appetitive lipping, an appetitive Pavlovian response that has been shown to reflect information about outcome value 34, 35, following presentation of that cue [36]. In other words, the intensity of the dopamine responses appears driven by reward information (indexed by lipping) rather than by changes in effort level. In line with these data, Pasquereau and Turner [30] also found little effect of upcoming energy expenditure on the activity of dopamine response, with only 13% of dopamine neurons encoding effort (compared to 47% for reward) (Figure 1A).

Although effort costs did influence performance parameters such as reaction times, it could be argued that this discrepancy between reward and effort coding came from the fact that the weights of the cost and benefit parameters on value were not completely equated, particularly because the willingness of the monkeys to work was only influenced by the effort costs on a small minority of sessions. However, a subsequent study by Varazzani *et al*. [18] also found that dopamine neurons were significantly more sensitive to information about upcoming reward size than about upcoming energy expenditure (Figure 1B). This occurred even though here the weight of reward and effort on the willingness to work of the monkeys was similar in magnitude (although opposite in direction), ruling out the possibility that the difference in sensitivity to reward versus effort in dopamine neurons is related to a lower behavioral sensitivity to effort. Because of this reduced sensitivity to effort costs relative to reward benefits, the responses of dopamine neurons in this task, in isolation, did not predict upcoming choices, in contrast to other tasks where the outcome value only depended upon reward information 16, 29.

## The Influence of Upcoming Effort on Dopamine Release

One possible reason for the limited evidence for effort encoding by dopamine, outlined above, is that the studies to date have mostly targeted neurons in the substantia nigra pars compacta, which projects to dorsal parts of the striatum. By contrast, manipulations of dopamine transmission in rodents has strongly implicated mesolimbic pathways from the ventral tegmental area (VTA), particularly to the core of the nucleus accumbens (NAcC), as being necessary to allow animals to select options requiring additional effort for greater reward over a lower-reward alternative [10]. At first glance, this might suggest that dopamine levels in the NAcC would be strongly sensitive to upcoming effort costs. However, an alternative, in accordance with the electrophysiological data, would be that dopamine levels might principally encode predictions of future reward to allow animals to determine what would be an appropriate level of effort to exert [5].

**Figure 2:**
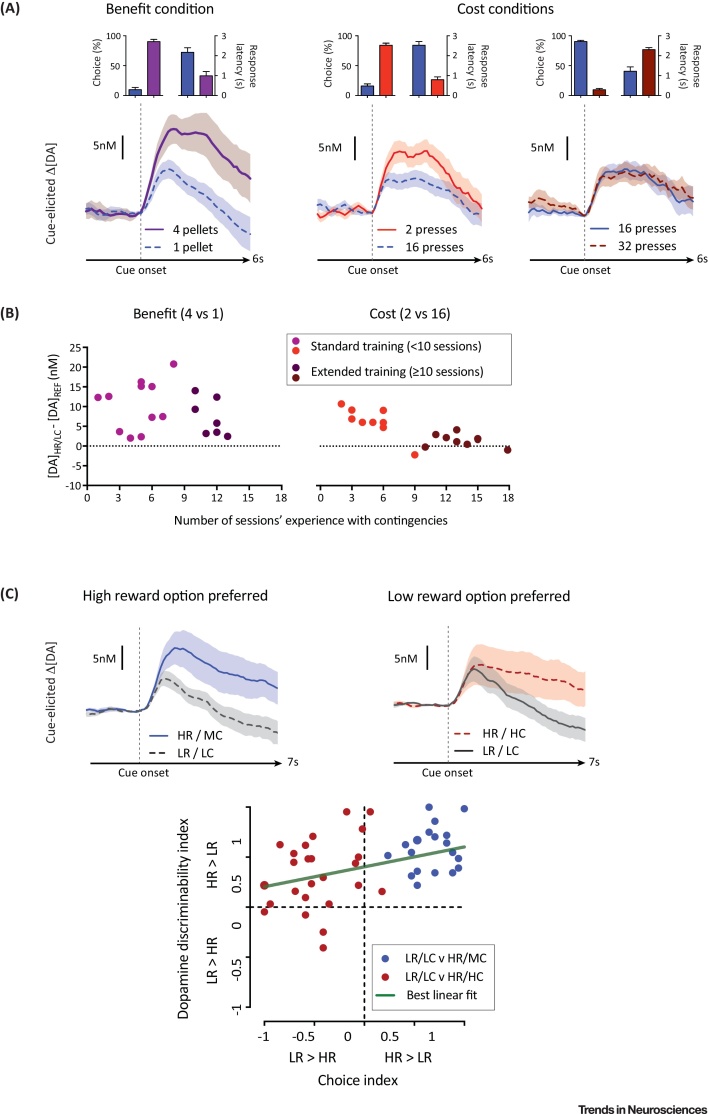
Cue-Elicited Dopamine (DA) Levels Are Primarily Modulated by Expected Future Benefits and Not by Anticipated Costs. (A) Behavior (choice performance and response latencies, upper panels) and cue-elicited dopamine levels (lower panels), recorded with fast-scan cyclic voltammetry in rat nucleus accumbens core, in conditions where rats were presented with options signaling availability of a future reward (food pellets) after paying an effort cost (repeated lever presses). In each condition there was one reference option (16 presses/1 pellet, blue bar/line) and one alternative that was associated with a higher benefit (16 presses/4 pellets, purple bar/line, left panels), lower cost (2 presses/1 pellet, red bar/line, mid panels), or higher cost (32 presses/1 pellet, burgundy bar/line, right panels). Filled lines correspond to the preferred option of each pair. (B) Difference in peak cue-elicited dopamine on higher benefit/lower cost trials ([DA]_HR/LC_) compared to reference trials ([DA]_REF_) in individual rats as a function of the amount of experience they had with those particular cost–benefit contingencies (standard training, <10 sessions of experience, lighter colored dots; extended training, ≥10 sessions of experience, darker colored dots). Although a greater peak dopamine was consistently recorded on higher-benefit trials regardless of training experience (left panel), the difference in dopamine between lower-cost and reference trials reduced with increasing experience of this condition (right panel). Specifically, after extended training with the lower-cost contingencies, there was no reliable difference in cue-elicited dopamine on lower cost and reference trials, even though rats still exhibited a strong preference for the lower-cost option and responded faster on lower-cost trials. Note that, unlike in some studies (e.g., [60]), the cost/benefit contingencies in the experiments depicted here reversed each day, and that these data come only from trials after animals had achieved a stable preference for the higher-benefit or lower-cost option in each session. Therefore, these results neither reflect learning (in early sessions) nor habitual responding (in later sessions). Panels adapted, with permission, from [19]. (C) (Upper panel, left) dopamine responses to cues signaling availability of either a low-reward/low-cost option (LR/LC: 1 reward/4 presses) or a high-reward/mid-cost option (HR/MC, 8 presses/4 rewards; left panel). (Upper panel, right) As in the left panel, but comparing responses to the LR/LC option to a HR/high-cost option (HR/HC, ≥32 presses/4 rewards). The cost–benefit contingencies were again reversed every few sessions, and dopamine data were collected from trials after animals had achieved a stable preference for the HR/MC (versus LR/LC) or LR/LC (versus HR/HC). (Lower panel) A ‘dopamine discriminability index’ plotted against average preference across a session, quantified using a ‘choice index’ (HR−LR choices). The dopamine discriminability index was based on the area under the receiver operating characteristic (auROC) classifying dopamine release as discriminable on HR from LR trials in each session. As can be observed, although the choice index spans the full range of values, the dopamine index is strongly positively skewed, showing that it was more common to classify dopamine release as greater on HR than LR trials irrespective of preference (blue dots: LR/LC versus HR/MC; red dots: LR/LC versus HR/HC). Panels adapted, with permission, from [20].

To test this, Gan and colleagues [19] used fast-scan cyclic voltammetry to measure subsecond fluctuations in NAcC dopamine levels in response to cues signaling the availability of one of two options. One option always required animals to pay a ‘reference’ effort cost for reward (16 lever presses for 1 food pellet), and the other either had a different associated effort cost (2 or 32 lever presses) or a different reward value (4 or 0 food pellets). Importantly, the reduced cost and increased reward levels were selected to confer on average equal utility, and therefore any differences in dopamine release between the cost or reward conditions cannot be attributed to differences in net value. Exactly like the activity of dopamine neurons, cue-elicited dopamine release consistently scaled with anticipated future reward. Importantly, despite having an equivalent influence on response latency, learning rates, and overall preference, changes in effort costs had substantially less impact on dopamine levels (Figure 2A).

However, it is not the case, as has been sometimes described, that there was no influence of effort costs on dopamine levels; the modulation by effort was present, but was weaker and transient (it was only present for costs that were less than the reference and in animals who had not undergone extensive training) (Figure 2B). As with neuronal activity, this meant that dopamine levels in isolation could not be used to predict effort-based choices.

A complementary picture emerges from a companion study by Hollon and colleagues [20]. Instead of one option being objectively of higher value than the alternative, here rats were faced with a decision between one low-effort cost/low-reward option and another higher-effort cost/high-reward alternative. In separate conditions, the effort cost associated with the high reward was altered such that in one condition the animals preferred overcoming the higher effort/high reward, whereas in the other the cost was increased until the average preference was for the low-cost/low-reward option. Average cue-elicited dopamine levels were significantly greater on high-reward than on low-reward trials. As before, there was also a small, consistent influence of effort costs on cue-evoked dopamine, particularly the initial cue-evoked response. Importantly, this influence was again much weaker than the expectation of future reward, meaning that, even in conditions where the rats preferred and responded faster to the low-reward option, average dopamine levels were still greater following presentation of the high-effort/high-reward option (Figure 2C).

## What Role Might Rapid Changes in Dopamine Play in Effort-Related Choice?

**Figure 3:**
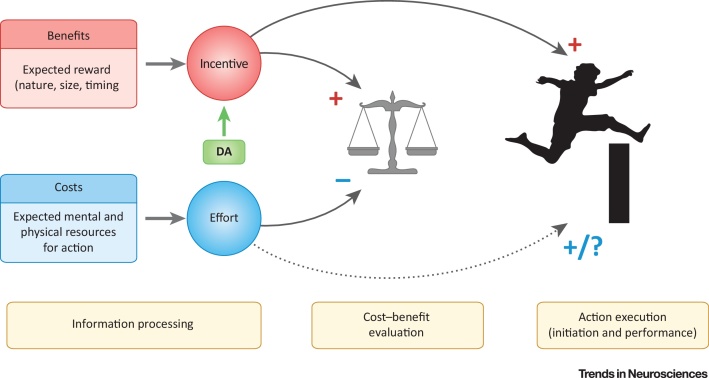
Key Figure: Influence of Expected Reward and Effort on Behavior

The emerging picture from the studies discussed above is one of convergence: dopamine is reliably modulated by expectations of future reward, whereas the negative influence of effort costs is much more limited and is only observed in restricted task conditions (Figure 3, Key Figure). What is remarkable is that such a consistent picture emerges despite differences in species, task, technique, and dopamine subsystem (nigrostriatal dopamine neurons versus mesolimbic dopamine release) (Box 3 for discussion of homogeneity and diversity of dopamine). Moreover, these results align with several other studies implicating dopamine more strongly in reward than in effort processing 37, 38, 39, 40. Thus, we believe that this feature is neither anecdotal nor due to an experimental artifact.

Box 3 **Is Dopamine Signaling a Single Functional Entity?**

For the sake of simplicity, we have chosen to treat the dopamine system as a functional unit. Indeed, our deliberate aim has been to focus on the concordance in the literature regarding dopamine and effort, highlighting the common features that were reliable enough to be described regardless of the species, the task, or the method used to measure activity. Nonetheless, we do not wish to underplay the widespread evidence for diversity of molecular features, anatomical connections, and coding across dopamine neurons. For instance:

(i) At the anatomical level, the projections of dopamine neurons vary as a function of their ventromedial to dorsolateral location in the midbrain and of their molecular properties 101, 102, 103. Note, however, that these are graded distinctions [104], and several studies point to interactions among these channels [105].

(ii) At a physiological level, a significant proportion of dopamine neurons within particular midbrain regions respond in a stereotyped way to rewards and their predictors 6, 28. However, dopamine neurons recorded in the distinct regions of the midbrain can also display distinct functional correlates, particularly concerning coding of reward and aversion (71, 106; but see also [107]). Moreover, there can even be heterogeneous activity patterns in subpopulations of dopamine neurons within midbrain regions 18, 30, 70. Such diversity is also apparent in terminal regions 72, 73, 108.

(iii) At a functional level, experiments have shown that artificial activation of dopamine neurons or their projections originating from distinct midbrain regions can have distinct functional consequences 109, 110, even if stimulation of both SNc and VTA can induce appetitive effects [111].

Although this evidence converges to suggest that the dopamine system can be broken down into several functional units, the exact boundaries between these units and the exact nature of their functions remain to be elucidated. Crucially, the level of description remains strongly dependent upon the question of interest and the scale at which it is studied (e.g., the information encoded by single dopamine neurons versus that communicated by diffusion-based volume transmission in terminal regions). A useful comparison may be with primary visual cortex which, for some purposes, can be considered as a functional entity even though it can be broken down into several patches based on ocular dominance or the position of the visual input, and is also part of an interconnected set of cortical regions involved in visual processing.

Alt-text: Box 3

Crucially, this holds true even though, behaviorally, the choices of the animals were strongly modulated by both effort and reward to a degree that was quantitatively equivalent. In other words, there is a clear uncoupling between cue-elicited dopamine activity and trial-by-trial effort-related decisions. Therefore, we would contend that dopamine cannot act as a ‘common currency’ that integrates across all economic variables to signal the net utility of an available option (or, for that matter, deviations from average expectation, i.e., a utility prediction error).

How then can one reconcile these data with the careful, elegant experiments showing a strong relationship between the activity of dopamine neurons and quantitative utility prediction error 32, 41? One way would be simply to assume that choices about whether or not to invest effort form a fundamentally distinct class of decisions that are separate from other cost–benefit scenarios such as intertemporal or risky choice (Box 2). In contrast to costs such as delay or probability, effort is much more closely aligned with action systems. Indeed, several studies have shown that the evaluation of effort costs involve cortical–striatal circuits closely related to response selection, such as dorsomedial frontal cortex (anterior cingulate and supplementary motor cortex) and dorsal striatum/putamen 42, 43, 44, 45, 46, 47, 48, and not circuits centered on orbital and ventromedial prefrontal cortices and parts of ventral striatum, which are more commonly implicated in benefits-based decisions (e.g., 49, 50). Therefore, decisions relying on a cost–benefit analysis would result from a joint influence of these two systems on motor output.

However, although it is indisputable that dopamine encodes parameters about expected future outcomes relevant to a decision, there is also increasing evidence that its function may not be to directly guide action selection between simultaneously available options. Indeed, even when choices are defined solely by differences in outcomes, midbrain dopamine activity and NAcC dopamine levels in response to individual options are not necessarily a reliable predictor of preference because they often signal the value of an upcoming choice even when it is not reward-maximizing 29, 51. From this perspective, dopamine seems to signal the potential benefits of a decision that has already been finalized.

What then might be the functions of transient increases in dopamine before effort-related actions? One possibility is that dopamine does not signal predictions of future reward to guide what action to take, but instead provides a signal to shape whether (and possibly also when and how fast) to act given the potential benefits of taking a presented opportunity in a particular environment. In naturalistic settings, potential rewards are often encountered sequentially rather than simultaneously. This implies that a key computation, recurring across species, is whether or not to engage with a presented opportunity [52]. Thus, we would argue that dopamine activation reflects the incentive influence of a potential reward on behavior that could lead to obtaining it (Figure 3). While such signals will tend to be elicited by external stimuli, they can nonetheless be contextually regulated by afferent input 53, 54, allowing control over when it is beneficial to engage versus when it is better to display restraint.

For instance, NAcC dopamine levels are dynamically modulated not only by reward prediction errors but also by whether or not an action should be made to gain the reward 55, 56. Similarly, a comparable phenomenon has been observed in the firing of dopamine neurons recorded from substantia nigra pars compacta in monkeys 18, 36, indicating that this phenomenon is again reliable across species, task, recording technique, and dopamine subsystem. Put another way, dopamine activity would reflect an instantaneous estimate of the change in average reward rate – or potential net energy gain, based on the internal state of the animal 57, 58 – that would be achieved by pursuing an option. This signal could, in turn, motivate a new course of action to be initiated [59]. Indeed, because effort costs can cause animals to act more slowly or perform less reliably, it is worth considering that what may appear in some experimental situations as an influence of effort on dopamine may, without careful controls (such as equating trial rates irrespective of high- or low-effort choices: compare [19] with [60]), actually relate to changes in reward availability.

We would speculate that this signal incorporates effort costs only if the pursuit of the reward has an influence on the net income or reward rate of the animal. This close connection with rates of reward might also explain the important link between dopamine and time judgments [61], and between dopamine and action vigor 37, 62, 63, given that decisions of when and how fast to act both will influence the rate of events. Of note, effort costs in laboratory paradigms are typically of relatively low energy demand and also tend not to induce large fluctuations of reward rate within an individual session. According to the framework outlined above, a natural consequence of these characteristics would be that recorded dopamine signals come to be dominated by predictions of future reward.

## Linking Recording and Manipulation Studies

The ideas discussed in earlier sections about the relationship between dopamine and effort emerged primarily from recent correlative recording studies in behaving animals. In addition, there is a history of studies examining the effects of pharmacological manipulations of dopamine transmission during effort-based decision making 2, 8, 10. However, it is not always straightforward to align the findings from recording studies with the pharmacological literature on this topic given the differences in timescale and approach. An important future step will be to combine recording and manipulation techniques, and to use manipulation techniques with better temporal resolution, to understand better how disrupting neurochemistry can in turn influence dopamine coding. Nonetheless, even given these caveats, we believe that the proposed role for dopamine outlined here can fit well alongside the existing literature on the effects of disrupting dopamine transmission.

A frequent observation, for instance, is that blocking dopamine receptors reduces the likelihood and speed of engagement as a function of future reward 9, 64, 65. This observation fits nicely into a picture where rapid increases in dopamine provide a unitary signal to different terminal regions about the potential net gain of a presented opportunity, and can thus help to authorize decisions about when to act and when not to act. The fact that we have observed such signals particularly clearly in situations when animals are required to switch from an inactive or disengaged state to an active one may also be related to a particular role for dopamine when needing to initiate a non-stereotyped response [3].

We have emphasized what we see as a consensus in the literature on the point that effort costs have a limited influence on dopamine activity. This may seem, at first glance, contradictory to the fact that pharmacological manipulations of dopamine often cause pronounced changes in allocation of effort. There are some subtleties here, however, that can perhaps easily be missed, particularly when comparing correlative and interventional approaches. One subtle distinction is that, even if dopamine can promote energy expenditure, it only does so as a function of the upcoming reward, and not as a function of the upcoming (energy) cost itself. Crucially, the few recent experiments that examined the influence of dopamine treatments in tasks where efforts costs and reward benefits were dissociated, also found a stronger influence on reward-based over effort-based decisions 37, 40. Therefore, even if these manipulations lack the temporal and anatomical precision of recording studies, they reinforce the idea that the dopamine system as a whole is much more sensitive to potential benefits than to potential effort costs, and demonstrate the generality of this relation, as well as its causal nature.

If effort costs are not directly encoded by dopamine signals, an obvious question remains: which circuits signal effort costs, and how these might interact with dopamine? As described earlier, there is general agreement that anterior cingulate cortex and dorsomedial motor areas play some role in representing effort, and the former has recently been shown to modulate VTA activity during an effort task [53]. When it comes to the question of what neural pathways allow effort costs to be overcome, one intriguing possibility is that, despite all the attention on dopamine, it may be that other neurochemical systems are crucial in this context. For instance, locus coeruleus neurons are strongly active both immediately before and after an effortful action is initiated 18, 36, suggesting that noradrenaline might be crucial to mobilize energy to overcome an effort cost. Some striatal cholinergic interneurons also increase their activity when engaging in a high-effort or small-reward trial [66]. Although serotonin has tended to be linked more closely to delay-based decisions, recent evidence suggests it may also affect how effort costs accumulate over time as well as the vigor of responding (the latter possibly via dopamine interactions) 67, 68.

## Concluding Remarks

The function of dopamine has long generated a great deal of debate, and will likely continue to do so. We highlight here what we believe to be a conspicuous point of consensus. Crucially, this consensus is only arrived at when the data are considered for what they specifically show, putting aside any attempt to fit them into one or other established position. To us, it seems that the data converge to a picture where dopamine signals display strong and consistent reward encoding, but limited and sometimes transient effort encoding.

Clearly, there are many issues that remain to be addressed (see Outstanding Questions) and we would be surprised if there are no new points of divergence once various aspects of effort and different dopamine pathways are examined in more detail. For instance, our starting position is that the convergence we observe suggests there may be a unitary, or at least general, function for dopamine in promoting action based on predictions of future benefits (*cf* [69] and [9] for complementary ideas). However, given ongoing debates concerning the degree of diversity of coding in midbrain dopamine neurons (e.g., 18, 30, 70, 71), as well as clear variations in patterns of release in terminal regions 72, 73, we are cognizant that there may be finer-grained distinctions to be drawn. In particular, it will be important to understand better how the role of dopamine in spontaneous movement and timing dovetails with some of our viewpoints 61, 63, 70, 74. Nonetheless, we believe that the potential to find correspondences across species, technique, and task shows that it should be possible to harness the advantages that the different approaches confer to uncover common and conserved functions of dopamine.

Outstanding Questions What is the timescale of dopamine action? Does the average background (‘tonic’) dopamine level have a role distinct from that of the transient cue-evoked signals in motivating animals to overcome effort, or has the distinction between its action at fast and slow timescales been overstated?

Does dopamine also influence movement parameters? NAcC dopamine levels do not straightforwardly correlate with movement times. However, several studies have suggested a link between nigrostriatal dopamine, at least, and the vigor with which an action is performed.

What is the relationship between dopamine and other structures, such as anterior cingulate cortex, and other neurochemical systems during effort-related choices?
